# Interfacial Bonding and Abrasive Wear Behavior of Iron Matrix Composite Reinforced by Ceramic Particles

**DOI:** 10.3390/ma12223646

**Published:** 2019-11-06

**Authors:** Yefei Li, Cong Li, Shuli Tang, Qiaoling Zheng, Juan Wang, Zhibo Zhang, Zhicheng Wang

**Affiliations:** 1 State Key Laboratory for Mechanical Behavior of Materials, Xi’an Jiaotong University, Xi’an 710049, China; yefeili@126.com (Y.L.); licong2150120060@163.com (C.L.); zhengql@mail.xjtu.edu.cn (Q.Z.); 2 Xi’an Microelectronic Technology Institute, Xi’an 710054, China; shulitangxjtu@126.com; 3 Guangdong Institute of Materials and Processing, Guangzhou 510650, China; sweetanna@foxmail.com; 4 Yancheng Jiuheng Industry & Trade Co., Ltd., Yancheng 224021, China; admin@ycjhgm.com

**Keywords:** iron matrix composite, ceramic particle, slurry coating, three-body abrasive wear

## Abstract

Using zirconia toughened alumina (ZTA) particles and Ni–Ti complex powders as raw materials, high-Cr cast iron reinforced by ZTA particles was prepared by an infiltration casting process. A continuous transition layer formed at the interface between ZTA particles and the Cr15 matrix, which proves that there is strong metallurgical interfacial bonding at the interface. The phases in the Ni–Ti layer of the ZTA_P_/Fe composite were preserved compared with the microstructure of sintered ZTA ceramic preform. The hardness of the Ni_3_Ti, TiO and AlNi_2_Ti phases in the interfacial transition layer was measured by the nano-indentation method, which is 12.5 GPa, 16.1 GPa and 9.2 GPa, respectively. The three-body wear resistance of the composite reached 12.6 times that of high-Cr cast iron.

## 1. Introduction

Hard ceramics have high hardness, advanced temperature stability, desirable stiffness and favorable corrosion resistance, which means they are widely used as cutting tools, wear resistant parts, aircraft components, etc. [1,2,3,4]. In particular, hard ceramics are extensively used as reinforcement for fabrication of particles reinforced iron matrix composites (PRIMCs). PRIMCs are of great industrial importance not only because the iron matrix is inexpensive, but also because the particles demonstrate superior properties of high elastic modulus, superior hardness and wear resistance [5,6,7,8]. PRIMCs are widely used in strong impact or high stress wear conditions, such as a hammer crusher, vertical mill grinding roller and grinding disc.

Among many PRIMCs, ZTA_P_/Fe composites have become a kind of promising wear resistant material, mainly because of the outstanding thermal physical and mechanical properties of ZTA ceramics [5]. Generally, the physical properties of ZTA varied over a relatively large range by modifying the proportion of ZrO_2_ for different working demands [9,10,11,12,13]. The difference in thermal expansion coefficient (TEC) between ZTA ceramic (7.8–20 × 10^−6^ °C^−1^ [5,14]) and iron matrix (9.2–16.9 × 10^−6^ °C^−1^ [15]) is lower than other hard ceramics, such as WC (3.8 × 10^−6^ °C^−1^ [5]), α-Al_2_O_3_ (6.4–7.5 × 10^−6^ °C^−1^ [16]), α-SiO_2_ (0.5–4.1 × 10^−6^ °C^−1^ [17]) and SiC (4.1–4.6 × 10^−6^ °C^−1^ [18]). The high hardness (12–20 GPa [19,20]) and special self-toughening property of ZTA enable the potential excellent wear resistance of ZTA_P_/Fe composites under severe wear conditions (e.g., high-stress wear condition, high impact wear condition, etc.).

However, the ZTA is hardly wetted by molten iron, and the mechanical bonding is easily formed at the interface between ZTA and iron; thereby the ceramic particles at the surface of the composite may be stripped under severe wear conditions. In order to improve the interfacial bonding property, Sui et al. [21] prepared a kind of ZTA_P_/Fe composite by an infiltration casting process; the TiO_2_ powder and Na_2_O·*n*H_2_O·SiO_2_ solution were coated onto ZTA particles, and an interfacial transition layer formed between ZTA particles and iron matrix after casting, which mainly contains amorphous Na_4_SiO_4_ phases. In our past work [22], we developed a nickel-plating process to deposit ZTA particles, which benefited the interfacial bonding behavior and therefore the three-body wear resistance. However, the interfacial bonding mechanism is still a mechanical bonding. By employing Ti and Si elements at the Al_2_O_3P_/Fe interface, Bahraini et al. [23] prepared a steel-based composite by using different infiltration atmospheres, where the chemical reaction could be observed in the interface. Vasic et al. [24] studied the chemical reaction between molten iron and Al_2_O_3_ ceramic plate deposited by Ti elements on the surface, and they found a large number of irregular Fe–Ti alloys formed at the interface between Al_2_O_3_ ceramic and iron. Inspired by the above studies, the Ni–Ti powder mixture, designed as an interfacial transition layer, was introduced into the interface of ZTA_P_/Fe composites in this work. The interfacial bonding characteristic between ZTA and iron was analyzed; the three-body abrasive wear test was performed, and its wear physical mechanism is proposed.

## 2. Materials and Methods

### 2.1. Preparation of Composite

The ZTA particles (~1 mm in diameter, containing ~80 vol.% ZrO_2_), were chosen as reinforcement for the high-Cr cast iron matrix composite. An infiltration process, summarized below, produced the ZTA_P_/Fe composites.

**Figure 1:**
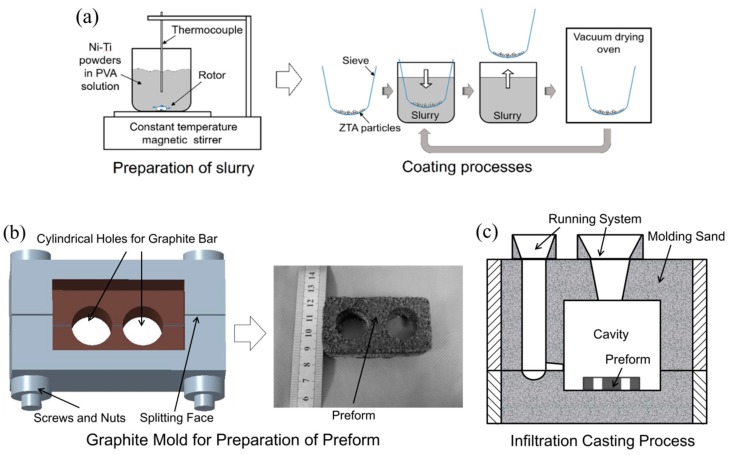
Schematic diagrams for preparation of the composite: (**a**) slurry coating process of ceramic particles; (**b**) graphite mold for preparation of preform; (**c**) infiltration casting process.

The first step was a slurry coating process, as shown in Figure 1a. This included: (i) Ni and Ti elemental powders (40–50 μm) were totally mixed in ethanol by alumina milling balls for 24 h. (ii) The slurry was prepared by dispersing the Ni–Ti powder mixture homogeneously in polyvinyl alcohol (PVA) solution by a constant-temperature magnetic agitator; PVA solution was feasible as slurry for various powders, such as Ti, Al, Fe, Ni, etc., because powders in PVA solution have excellent dispersibility and good stickiness [25,26,27,28]. (iii) The mesh with ZTA particles inside was dipped into the slurry. (iv) The mesh with ZTA particles inside was taken out of the slurry and dried in a vacuum drying oven at 100 °C. (v) Procedures (iii) and (iv) were repeated several times so that the coatings on the particles could be obtained.

The second step was preparation of the preform, which was prepared from coated ZTA particles by sintering them in a graphite mold with protection from an argon atmosphere. The sintering temperature was chosen as 1500 °C, which was slightly higher than the melting point of Ni (1428 °C). The holding time of sintering was 1 h because the preform can withstand the impact of the molten iron during the subsequent infiltration casting process. Normally, we evaluate the anti-impact of liquid iron by performing a compressive test, and so the compressive strength of the preform was obtained by a universal mechanical testing machine.

Lastly, the composite was prepared by an infiltration casting process as shown in Figure 1b. The preform was located at the bottom of the cavity before pouring the molten Cr15 matrix. The chemical compositions of which were: Cr 15 wt.%, C 3.1–3.2 wt.%, Si 0.5–1.0 wt.%, Mn 0.6–0.7 wt.%, Mo 1.0–1.5 wt.% and balanced Fe. The pouring temperature of the Cr15 matrix was 1550 °C, which was a little higher than common industrial practice (~1420 °C [29]) in order to improve the flowability of the molten iron. The casting composite ingot was shaken out from the sand mold after 12 h to avoid the foundry cracks.

### 2.2. Three-Body Abrasive Wear Tests

**Figure 2:**
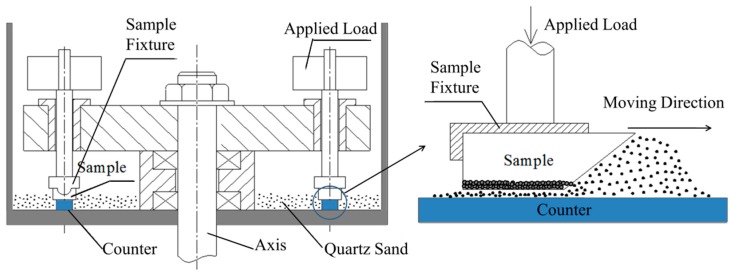
Diagrams of the three-body abrasive wear specimen and corresponding wear principle for the tester.

The three-body abrasive wear property of the composite, as well as reference Cr15 specimen, was tested by the MMH-5 block-on-counter abrasive wear tester (Hesion, Jinan, China) (Figure 2). All specimens were cut to a size of 15 × 25 × 6 mm, with a 45° slope forward in order to assist the abrasives to move between the specimens and counter, i.e., the contact area was 15 × 25 mm. The specimens were clamped, as shown in Figure 2, on an annealed AISI1020 steel counter (Hesion, Jinan, China) (~340 mm inside diameter), and were covered by loose SiO_2_ abrasives with ~150 μm average diameter and 5 kg in weight. The cross sectional dimension of the counter was rectangular (20 × 10 mm). The wear behavior was studied under loads of 1, 2 and 3 kg.

Firstly, a pre-wear process was executed for 60 min with 30 r/min in order to keep the similar surface condition of each specimen. The weight of the specimen (after cleaning in ethanol, similarly hereinafter) was measured and recorded as *m*_0_. After that, the three-body wear test was performed for 2.5 h with the same wear velocity. Three repetitions were made for each wear condition. The weight of each specimen was measured every 30 min by using a balance with an accuracy of 10^−4^ g, and recorded as *m*_1_, *m*_2_, *m*_3_, *m*_4_ and *m*_5_, respectively. Therefore, the mass loss of each duration Δ*m_i_* was calculated by Equation (1). The composite layer in the casting ingot consists of two parts, i.e., ZTA ceramic particles and iron matrix, which have different densities. The wear volume loss Δ*V_i_* is more effective than mass loss Δ*m_i_* at characterizing the wear behavior. The wear volume loss Δ*V_i_* can be found by using Equation (2).

(1) $$\Delta m_{i}=m_{i}^{}-m_{i-1}\;\;\;\;\left(I=\;1,\;2,\;\ldots ,\;5\right)$$

(2) $$\Delta V_{i}=\frac{\Delta m_{i}}{\alpha \cdot \rho_{p}+(1-\alpha )\cdot \rho_{m}}$$

where *α* refers to the volume fraction of ZTA particles in the composite layer of the casting ingot, given by the image analysis of the composite surfaces; *ρ_p_* and *ρ_m_* (in kg·m^−3^) refer to the densities of ZTA ceramic and Cr15, respectively. At last, in order to have an accurate evaluation, the relative wear resistance was specified by ε(=Δ*V_0_*/Δ*V_i_*), where Δ*V_0_* denotes the volume loss of reference Cr15 matrix.

## 3. Results and Discussion

### 3.1. The Structure of ZTA Ceramic Preforms

**Figure 3:**
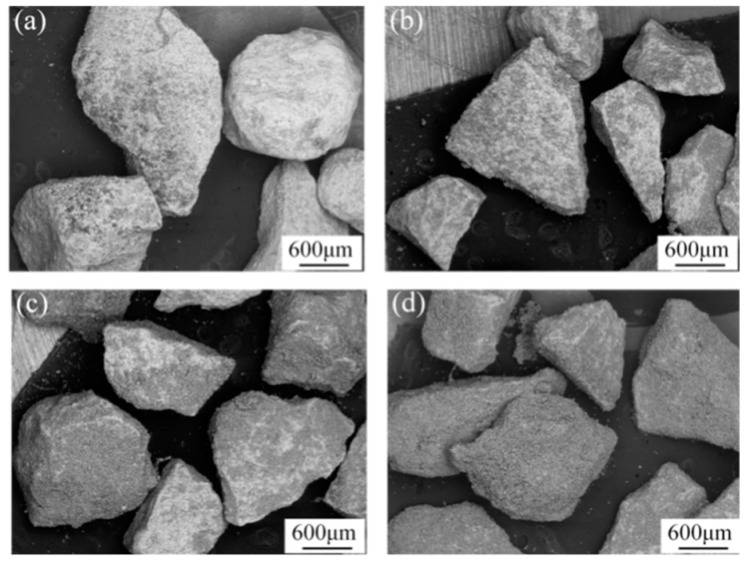
Macrographs of ZTA ceramic particles after different slurry coating times: (**a**) 5 times; (**b**) 10 times; (**c**) 15 times; (**d**) 20 times.

**Figure 4:**
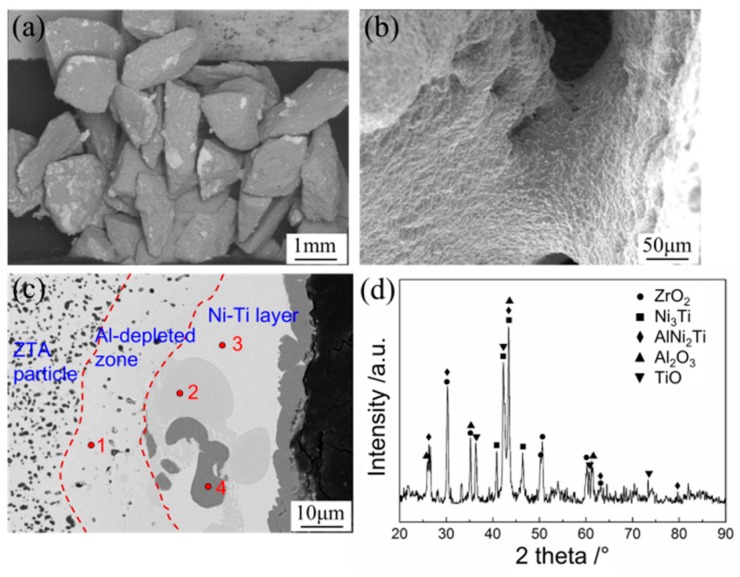
Surface morphologies of coated ZTA particle preform (**a**), sintering neck between two ZTA particles (**b**), local magnified microstructure of sintered coating (**c**), and XRD analysis results of sintered particles (**d**).

Figure 3 shows the surface morphology of coated ZTA particles under different coating times. It can be seen that the coating area of Ni–Ti powders on ZTA particles increased with the coating times, turning from bright to dark; both the weight of coated particles and the thickness of surface coating increased linearly with increasing coating time. After coating 20 times, the ZTA particles were covered by Ni–Ti powder. However, the surface coating was loose and porous, as the PVA solution in slurry was boiled-off in a vacuum drying oven. Thereafter, the coated ZTA particles were sintered under 1500 °C for 1 h. The morphology of sintered ZTA ceramic preform (Figure 4a,b) shows that all particles were connected and formed a porous structure, which will benefit the following cast infiltration process. Meanwhile, it can be seen that a compact sintered coating existed around ZTA ceramic particles and the metallic sintered necks connected the surrounding ZTA ceramics. The magnified image of the surface of sintered ZTA particles is shown in Figure 4c; the sintered shell covered the surface of the ZTA particles. Furthermore, the sintered ceramic particles were pulverized into a powder for X-ray diffraction analysis; the results are shown in Figure 4d, which indicate that the sintered ZTA particles consisted of multiple phases, i.e., ZrO_2_, Al_2_O_3_, Ni_3_Ti, AlNi_2_Ti and TiO. Among the above phases, Ni_3_Ti, AlNi_2_Ti and TiO were the reaction products between Ni–Ti coating and ZTA particles during sintering.

When sintered at 1500 °C, the nickel melted, which could increase the contact area between the Ni–Ti coating and ZTA particles. The Al atoms in ZTA particles diffused outside and reacted with the Ni–Ti coating, which formed the AlNi_2_Ti phase, and therefore, an Al-depleted zone was formed surrounding the ZTA particles as shown in Figure 4c. Outside the Al-depleted zone, a compact Ni–Ti layer was formed. For understanding the phase structure around ZTA particles, a semi-quantitative EDS analysis was performed and the results are listed in Table 1. The ZTA particle is on the left side of the dashed line in Figure 4c, and was composed of ZrO_2_ and Al_2_O_3_. In the middle Al-depleted zone, the ZrO_2_ phase was mainly found. In the right Ni–Ti coating zone, three different phases were detected, including AlNi_2_Ti (point 2), Ni_3_Ti (point 3) and TiO (point 4). The main phase in sintered coating is Ni_3_Ti, which is the most thermodynamically favorable among all Ni–Ti compounds [30]. The outmost ZTA particle preform was covered by TiO film because of the high chemical activity of Ti atoms. At the same time, the compressive strength of the preform was tested and the result was about 4.1 MPa, as the metallurgical bonding between the Ni–Ti coating and ZTA particles could improve the mechanical strength of the sintered ceramic preform.

### 3.2. Analysis of Composite Interface

**Figure 5:**
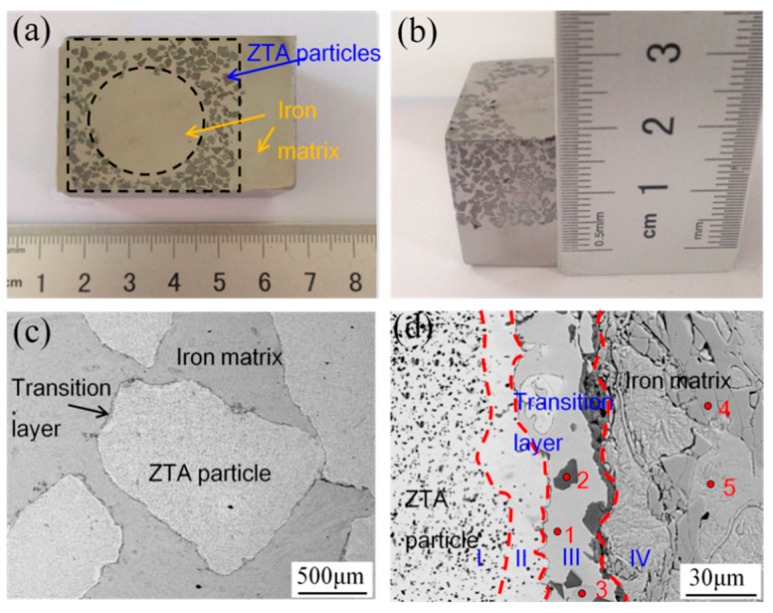
The microstructure of ZTAP/Fe composites: (**a**) distribution of ZTA particles, (**b**) cross section of infiltration layer, (**c**) the interfacial morphology, and (**d**) the high resolution interfacial structure.

Figure 5a,b show the segment of the ZTA_P_/Fe composite. It can be seen that the preform kept the original rectangular shape (with a circular hole) without destruction (Figure 5a) and the thickness of the composite layer reached 15 mm (Figure 5b). The circular hole in the previous preform will benefit the infiltration casting of liquid iron. This indicates that the strength of the preforms in the cavity is high enough to stand against the impact of molten iron during the mold filling processes of castings.

**Figure 6:**
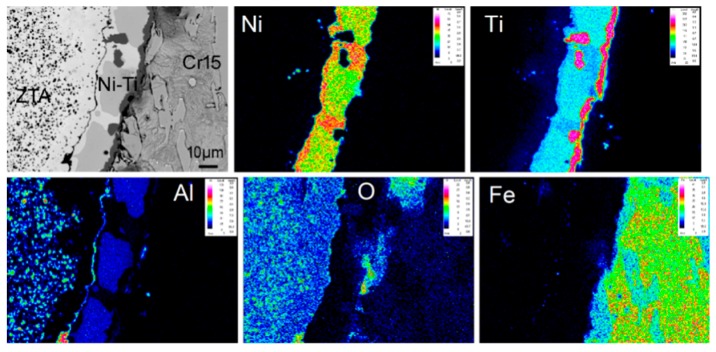
The elemental mapping results of interfacial phases by EPMA.

The interfacial microstructure of the composite is shown in Figure 5c,d. A continuous transition layer can be observed between the ZTA particles and the Cr15 matrix. Under high magnification (Figure 5d), it can be seen that around ZTA particles, four different zones existed, which ensure strong metallurgical bonding in the interface. Starting from the ZTA particle and moving outwards, the first zone was the original ZTA particle, while the second zone was an Al-depleted ceramic zone with much lower content of the Al_2_O_3_ phase than the first zone. In the third zone, the EDS analysis results, shown in Table 1, together with the EPMA results (Figure 6), illustrate that the main metallurgical phases were Ni_3_Ti, TiO and AlNi_2_Ti (point 1, 2, and 3 in Figure 5d, respectively). This indicates that the phases in the Ni–Ti layer did not change after the infiltration casting process compared with the sinter ZTA particle preform.

**Figure 7:**
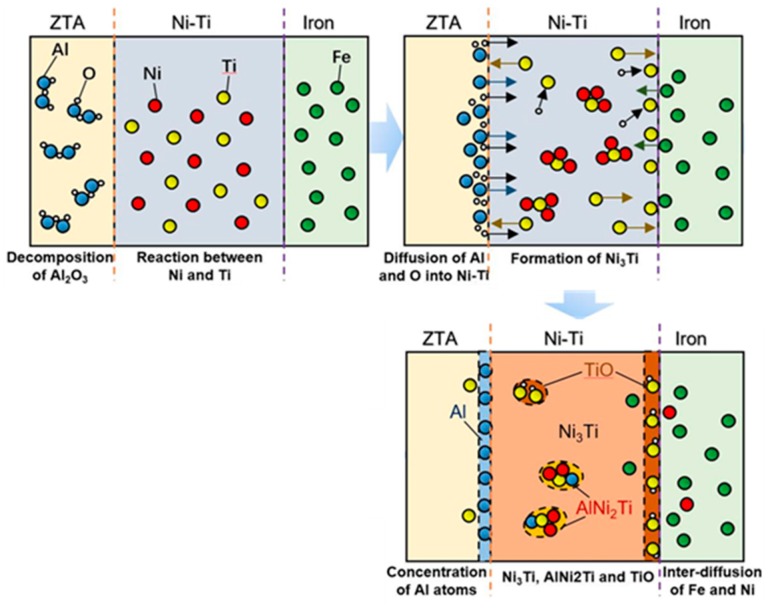
The formation mechanism at the interface of ZTA_P_/Fe composites.

The elemental mapping results of EPMA at the interfacial area are shown in Figure 6. Based on the elemental diffusion results in Figure 6, we proposed a formation mechanism model at the interface of ZTA_P_/Fe composites, as shown in Figure 7. It can be seen clearly that Al_2_O_3_ on the surface of the ZTA particle decomposed, and thereby the Al and O atoms diffused into the Ni–Ti layer. As a result, the Al element reacted with the Ni–Ti layer and formed a Ni–Ti–Al ternary compound. Furthermore, Ti atoms concentrated on the Ni–Ti layer/Cr15 interface and some O atoms existed at the Ti-rich zone, which formed TiO. Therefore, the transition layer consisted of continuous Ni_3_Ti, block-shape AlNi_2_Ti and a TiO zone just next to the Cr15 matrix. In addition, a small amount of Fe was also observed in the Ni–Ti layer due to elemental diffusion as illustrated by Figure 6.

**Figure 8:**
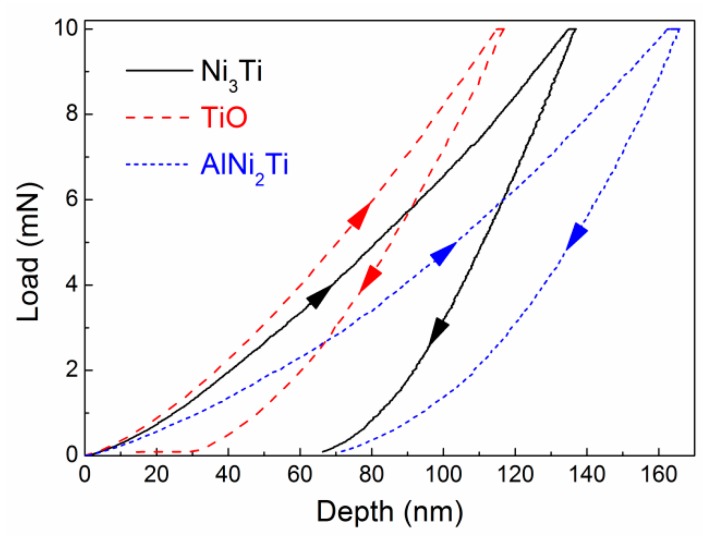
Typical load-depth curves of interfacial phases measured by thenano-indentation method.

Moreover, the mechanical property of interfacial phases has a significant effect on the interface property and thereby the wear behavior of the composite. In this work, the microhardness of Ni_3_Ti, TiO and AlNi_2_Ti in the interface was measured by the nano-indentation method. Figure 8 shows the typical load-depth curves of interfacial phases measured by the nano-indentation method, based on which we obtained the Young’s modulus and Vicker’s hardness, as listed in Table 2. The Vicker’s hardness of Ni_3_Ti, TiO and AlNi_2_Ti was 12.5 GPa, 16.1 GPa and 9.2 GPa, respectively. These testing values are very close to the theoretical hardness reported in [30,31,32,33,34,35]. The hardness of these interfacial phases are much higher than common white cast iron (~5.5 GPa), but lower than the ZTA ceramic (12–20 GPa [19,20]). Therefore, we suppose that the mechanical properties of the ZTA_P_/Fe composite would be improved by the interfacial transition phases (Ni_3_Ti, TiO, and AlNi_2_Ti).

### 3.3. Wear Resistance of the Composite

**Figure 9:**
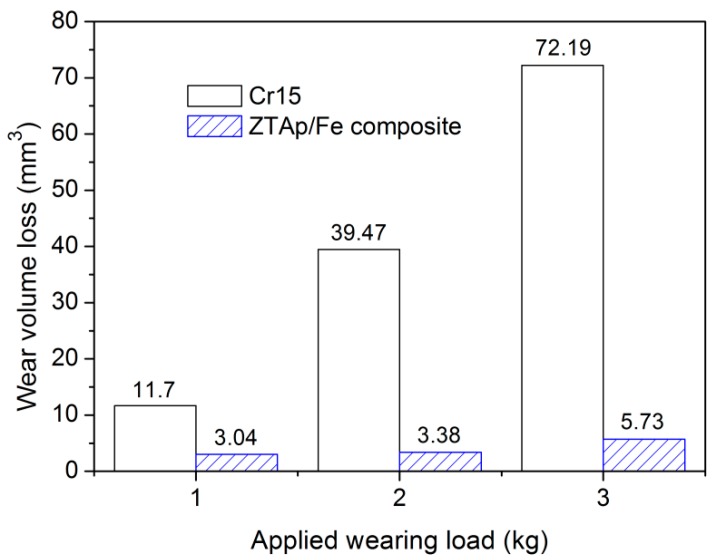
The average wear volume loss of each specimen under different applied loads.

The results of the three-body abrasive wear tests are shown in Figure 9. It is apparent that the wear volume loss of the ZTA_P_/Fe composite was much lower than Cr15 high Cr cast iron after a 2.5 h test. With increasing load from 1 kg to 3 kg (26.1 kPa to 78.4 kPa), the wear volume losses of the Cr15 matrix increased dramatically, while for the ZTA_P_/Fe composite, the increase rate of wear volume losses was much lower. Under 3 kg wear load, the wear resistance of the composite reached 12.6 times that of the Cr15 matrix, which indicates that the ZTA_P_/Fe composite showed excellent wear resistance, especially under severe wear conditions.

**Figure 10:**
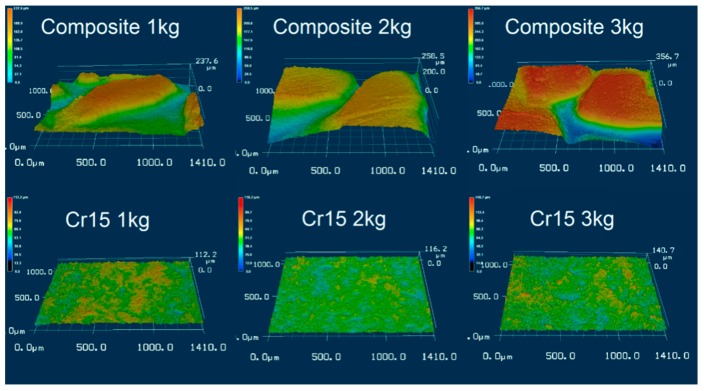
The laser scanning confocal topography (LSCT) of the worn surface of each specimen under different applied loads.

**Figure 11:**
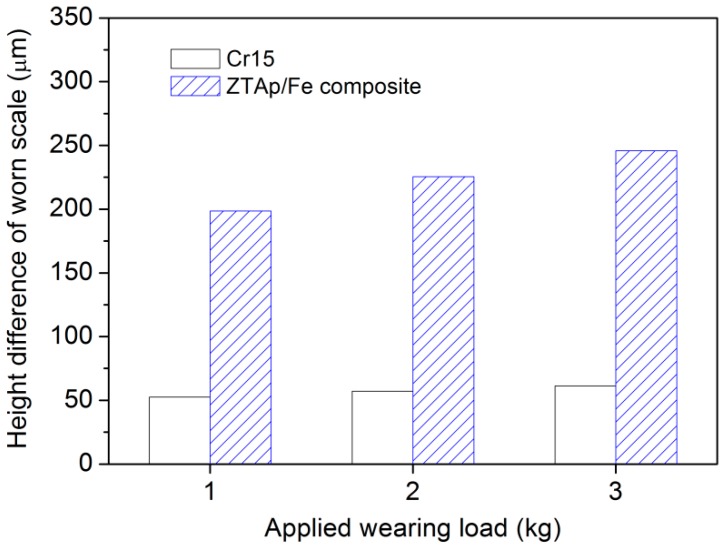
The height difference of the worn scale measured by laser scanning confocal topography for each specimen under different applied loads.

As illustrated in Figure 10, the worn surface morphology can be observed by the laser scanning confocal topography technique, the corresponding height difference of the worn scale for each tested specimen under different loads is shown in Figure 11. For composites at different wearing loads, the height of the iron matrix was low, which contributed to its deficient wear resistance, while the height of ZTA particles was high owing to its outstanding wear resistance. From Figure 11, at 1 kg wearing load, the ZTA particles protruded above the surrounding iron matrix in composites, with an average height of ~200 μm, compared with the height difference of ~50 μm for the Cr15 specimen. With increasing wearing load, the average height between ZTA particles and the surrounding matrix increased. The thickness reached ~250 μm at 3 kg wearing load. While for the Cr15 specimen, the height difference seemed more or less similar under each wearing load.

**Figure 12:**
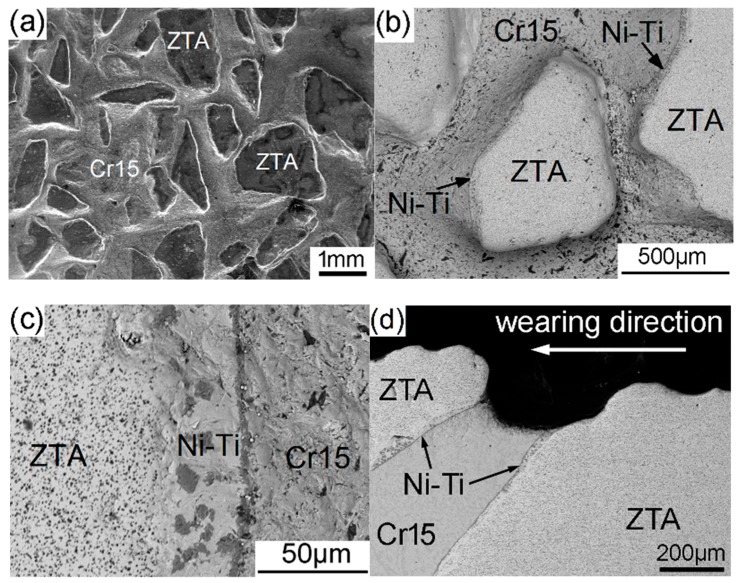
The secondary electron images of the composite after the wear test under the 3 kg load: (**a**) morphology of the worn surface; (**b**) protrusion of ZTA particles; (**c**) translation layer of the worn surface; (**d**) sub-surface microstructure.

Figure 12a,c show the worn surfaces of composite specimens after three-body abrasive wear tests under a 3 kg load. ZTA particles extruded gradually from the worn matrix owing to its higher hardness than the Cr15 matrix. The wear resistance of the composite improved significantly, because the protruded ZTA particles can bear the main wear force by abrasives and protect the surrounding iron matrix from abrasive wear. At the same time, ZTA particles did not peel off from the matrix, which indicates that the ZTA particles were tightly bonded by the transition Ni–Ti metallic layer and surrounding iron matrix. Meanwhile, ZTA particles in the composite were hardly broken or fractured even under a 3 kg load owing to the excellent toughness of the ZTA ceramic with high content of ZrO_2_ as shown in Figure 12c.

In order to clarify the wearing behavior of the composite more clearly, the sub-surface microstructure was observed on the cross sections of the composite after three-body abrasive wear tests under a 3 kg load. As observed in Figure 12, the typical hard carbides Cr_7_C_3_ inside the Cr15 matrix could hardly resist the abrasive wear of SiO_2_ due to their small size and high brittleness, so the Cr15 matrix part was severely worn. Cracks formed easily when the chromic carbides were crushed and fractured as shown in Figure 12b,c.

**Figure 13:**
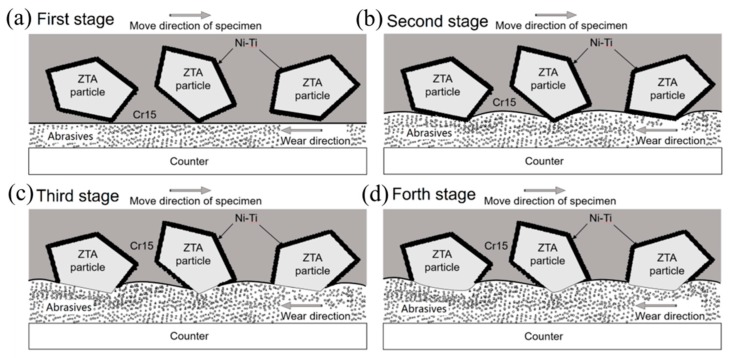
The physical mechanism of the three-body abrasive wear mechanism of ZTA_P_/Fe composites: (**a**) the first; (**b**) second; (**c**) third; and (**d**) fourth period of the wear test. The arrows represent the direction of the wear test.

The three-body abrasive wear mechanism of ZTA_P_/Fe composites is shown in Figure 13. At the beginning of the wear test, ZTA particles and matrix were worn simultaneously. Considering that the hardness of ZTA ceramic is higher than iron, the ductile Cr15 matrix was severely worn and concaved gradually, and therefore, the height difference between ZTA and iron became larger and larger; and finally, while keeping an almost constant value, at this condition the ZTA particles bore the main stress and protected iron from further wearing. Meanwhile, the iron matrix bonded ZTA ceramic tightly with the help of the Ni–Ti intermetallic layer, which prevented the ZTA particle from peeling off. Actually, the combination of the strong bonding property between ZTA ceramic and iron, and the protecting effect of ZTA ceramic on iron matrix, gave the composites an advanced wear resistance [36]. The ZTA_P_/Fe composites show great potential to substitute for traditional wear resistant material and be widely used in industrial applications.

## 4. Conclusions

The porous ZTA particles preform was prepared by sintering in a vacuum under 1500 °C. The strength of the preform reached 4.1 MPa because of the metallic sintered shell and necks connecting the ZTA particles. The ZTA_P_/Fe composites were fabricated by an infiltration casting process and the thickness of the composite layer reached 15 mm. The interface of the ZTA_P_/Fe composites showed metallurgical bonding. A Ni–Ti transition layer consisting of Ni_3_Ti, TiO and AlNi_2_Ti existed between the ZTA particles and the iron matrix. The measured hardness was 12.5, 16.1 and 9.2 GPa for Ni_3_Ti, TiO and AlNi_2_Ti, respectively. The wear resistance of ZTA_P_/Fe composites remarkably improved with increasing load and reached 12.6 times the reference Cr15 specimen. This is mainly because the interface was tightly bonded by a transition Ni–Ti layer; and the protruded ZTA particles can bear the main wear force by abrasives and protect the surrounding iron matrix from further failure.

## Figures and Tables

**Table 1:** Energy dispersive spectrometer (EDS) analysis of the points at ceramic preforms and composite interfaces (at.%).

Point to Be Analyzed		Element								
		Ni	Ti	Al	O	Zr	C	Fe	Mn	Cr
Figure 4c	Point 1		3.12		53.54	43.34				
	Point 2	56.57	22.67	20.76						
	Point 3	74.39	25.61							
	Point 4	0.75	86.07		13.18					
Figure 5d	Point 1	71.56	13.86	8.73			0.22	4.90		0.73
	Point 2	1.42	54.83		42.87		0.88			
	Point 3	54.28	14.56	17.22			0.16	9.41	1.66	2.71
	Point 4						1.04	43.55	1.81	53.60
	Point 5	0.76					0.51	85.83	4.43	8.47

**Table 2:** Micro mechanical properties of interfacial phases measured by thenano-indentation method. The values in parenthesis refer to DFT results calculated by this work.

Phases	Young’s Modulus/GPa	Micro-Hardness/GPa
Ni_3_Ti	211 (253^a^, 260^b^)	12.5 (10.5^a^)
TiO	225 (267^c^)	16.1 (14.3^d^)
AlNi_2_Ti	165 (178.1^e^)	9.2 (8.1^f^)

^a–f^: Theoretical data from [30,31,32,33,34,35].
